# Kinship Index Variations among Populations and Thresholds for Familial Searching

**DOI:** 10.1371/journal.pone.0037474

**Published:** 2012-05-16

**Authors:** Jianye Ge, Bruce Budowle

**Affiliations:** Department of Forensic and Investigative Genetics, Institute of Applied Genetics, Health Science Center, University of North Texas, Fort Worth, Texas, United States of America; University of California Irvine, United States of America

## Abstract

Current familial searching strategies are developed primarily based on autosomal STR loci, since most of the offender profiles in the forensic DNA databases do not contain Y-STR or mitochondrial DNA data. There are generally two familial searching methods, Identity-by-State (IBS) based methods or kinship index (KI) based methods. The KI based method is an analytically superior method because the allele frequency information is considered as opposed to solely allele counting. However, multiple KIs should be calculated if the unknown forensic profile may be attributed to multiple possible relevant populations. An important practical issue is the KI threshold to select for limiting the list of candidates from a search. There are generally three strategies of setting the KI threshold for familial searching: (1) SWGDAM recommendation 6; (2) minimum KI≥KI threshold; and (3) maximum KI≥KI threshold. These strategies were evaluated and compared by using both simulation data and empirical data. The minimum KI will tend to be closer to the KI appropriate for the population of which the forensic profile belongs. The minimum KI≥KI threshold performs better than the maximum KI≥KI threshold. The SWGDAM strategy may be too stringent for familial searching with large databases (e.g., 1 million or more profiles), because its KI thresholds depend on the database size and the KI thresholds of large databases have a higher probability to exclude true relatives than smaller databases. Minimum KI≥KI threshold strategy is a better option, as it provides the flexibility to adjust the KI threshold according to a pre-determined number of candidates or false positive/negative rates. Joint use of both IBS and KI does not significantly reduce the chance of including true relatives in a candidate list, but does provide a higher efficiency of familial searching.

## Introduction

DNA based familial searching is an indirect way to develop investigative leads of the donor of an evidence sample by identifying close biological relatives (e.g., parents, children, full-sibs) in a DNA database. This approach has been used successfully to identify perpetrators of crimes in a number of cases [1]. There are two general methods proposed for familial searching, Identity-by-State (IBS)-based or Kinship Index (KI)-based [2]–[13]. The IBS-based method compares the number of shared alleles or loci between the forensic profile and the offender profile(s) in a criminal database. Potential candidate relatives of the source of the forensic profile are indicated if the number of shared alleles or loci reaches a predefined threshold. This method is simple, fast and relatively easy to implement. However, the ancestry information of the profiles and allele frequency data are ignored. In contrast, the KI-based method compares the joint probabilities of the forensic and offender profiles given that the donors are related (e.g., parent-child or full-sib) versus they are unrelated. In the KI-based method, either KI value or database size adjusted KI measure, such as EKR (i.e., EKR = KI/N) [7], may be used as a cut-off threshold measure for generating a list of candidates. Although KI-based methods are superior to an IBS method, multiple KIs for multiple populations have to be considered in familial searching, since criminal databases, such as those in the US, do not contain ethnic origin information and the population affinity of the forensic profile is typically unknown. Little direction has been provided on determining what threshold(s) should be set with multiple KIs for selecting candidates.

Karlsson et al. [14] have shown that the KI could substantially vary among the populations. Ge et al. [11], using simulation methods, also found that a good proportion of KIs can vary more than 100 fold for the four major US populations, and the variation increases with additional populations. Attempting to address the KI variation among reference populations, SWGDAM's [7] recommendation 6 suggested that the maximum and minimum EKRs among Caucasians, African Americans, southwestern Hispanics, and southeastern Hispanics should be greater than 1 and 0.1, respectively. However, no validation data were provided to evaluate the effectiveness of the recommendation. California implemented this recommendation for three populations instead of four (i.e., southeastern Hispanics was excluded) and evaluated the false negative and false positive rates for 100 test families in a database with 1 million profiles [10]. A more comprehensive study with a larger number of pedigrees and various sizes of databases, such as from small local databases (∼10,000) to national databases (∼10 million), would have provided more insight on the performance (in terms of false positive and false negative rates) of recommendation 6.

The cut-off threshold also can be set at the minimum or maximum KI among the populations, i.e., potential candidate relatives of the source of a forensic profile are suggested if the minimum or maximum KI reach the cut-off threshold. For example, suppose the cut-off KI threshold is set at 200 and the KIs (for full-sib relationship) of a forensic profile and an offender profile are 100, 200, 50, and 500 for the four major US populations, this offender profile is excluded using the minimum KI strategy since the minimum KI among the populations (i.e., 50 and 100) is less than the threshold, or the offender profile is included using the maximum KI strategy since the maximum KI (i.e., 500) is higher than the threshold. Apparently, the minimum KI strategy is more stringent than the maximum KI strategy. This maximum KI criterion could have misleading consequences as there is a higher probability that the lowest KI represents the true population affinity of the profile/candidate.

In this study, the minimum KI and maximum KI are compared with the true KI to determine which value better reflects the true KI. Then, the three primary KI variation cut-off strategies for familial searching are compared: (1) SWGDAM recommendation 6; (2) minimum KI≥KI threshold; and (3) maximum KI≥KI threshold. These strategies will be evaluated in terms of their false positive and false negative rates through simulation studies and empirical data. Finally, the KI threshold strategy for multiple populations is discussed.

## Methods

The same likelihood ratio and simulation methods as described by Ge et al. [11] were used. The likelihood ratio based method basically calculates the pairwise kinship ratio or KI for the forensic profile (*X*) and the candidate profile (*Y*), as shown in Equation 1, where Pr(X,Y|Relationship) is the probability of observing the two genetic profiles for a given relationship [11]. For a father-child relationship, the KI is the Paternity Index (PI). MPKin [15] was used to calculate the KI.
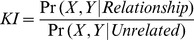
(1)


One million DNA profile pairs were simulated for each relationship (i.e., unrelated, parent-child, and full-sib) with 13 CODIS STR loci population data [16], [17] from each major population (i.e., Caucasian, African American, southwestern Hispanic, and southeastern Hispanic) using MPKin [15]. To generate simulated data, the genotypes of founders (i.e., individuals without parents in the pedigree) were randomly assigned according to the genotype frequencies and each locus was treated independently. For simplicity, this analysis did not include population substructure (i.e., Fst = 0). Founders transmitted with equal probability a single allele at each locus to his/her offspring.

Profile pairs were simulated using one population and then the KIs of each pair were calculated using all four US major populations. For simplicity, population substructure and mutation were ignored in these simulations although MPKin allows both factors in simulation, because the effects of population substructure and mutation are generally minor [11]. In addition, KI values of 112 African, 134 Caucasian, and 121 southwestern Hispanic true mother-child pairs from paternity testing cases were calculated. The mother-child relationships were reported by the mother and confirmed with CODIS loci genotyping yielding a minimum KI of at least 1,000 for four major populations (i.e., Caucasian, African American, southwestern Hispanic, and southeastern Hispanic).

## Results

**Figure 1:**
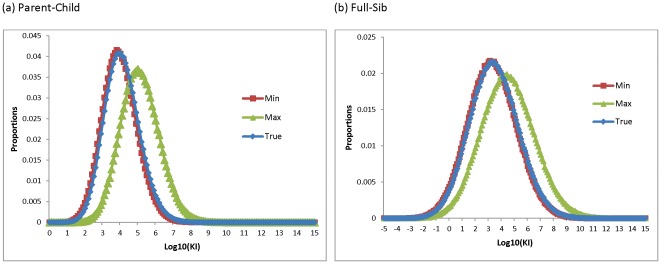
Distributions of the minimum KI, the maximum KI, and the true KI for (a) Parent-Child and (b) Full-Sib relationships with 13 CODIS core loci. The related pairs were simulated with Caucasian data, and KIs were calculated with African American, Caucasian, southeastern Hispanic, and southwestern Hispanic population data.

The distributions of the minimum KI, the maximum KI, and the true KI for Parent-Child and Full-Sib relationships with the 13 CODIS core loci were compared (Figure 1). The related pairs were simulated with Caucasian data, and the KIs were calculated using African American, Caucasian, Southeast Hispanic, and Southwest Hispanic population data. The distributions of the true KI were closer to those of the minimum KI than to the maximum KI. Similar results were observed for the two-person pedigrees generated using other reference profiles calculated with different population data. For relationships (i.e., profiles) generated by Caucasian population data, about 59.1% and 62.9% of true Parent-Child and Full-Sib pairs had the minimum KI belonging to the true population (e.g., Caucasian) (Table 1); about 23.1% and 21.8% of the second lowest KI belong to the Caucasian group for true Parent-Child and Full-Sib pairs, respectively. Similar accuracies were observed for the African American and southwestern Hispanic groups. The southeastern Hispanics had relatively low accuracies because allele frequencies of southeastern and southwestern Hispanics were similar and ∼25% of the KIs of southeastern pedigrees had the minimum KIs with the southwestern Hispanic population. The average heterozygosities of the 13 CODIS loci are 0.766 and 0.783 for the southwestern Hispanics and the southeastern Hispanics, respectively. This difference supports that the southwestern Hispanics have more common alleles than the southeastern Hispanics. Thus, generally, lower KIs were obtained with the southwestern Hispanic population data than with southeastern Hispanic population data. Higher accuracies for the minimum KI likely would be obtained if the Hispanic populations were merged. Based on the observations in Figure 1 and Table 1, the minimum KI is on average likely to be closer to the true KI, which is consistent with results of single source profile affinity with a population [18]. There are two explanations for this observation. First, the average profile tends to have common alleles with higher allele frequencies in the true population and may have lower allele frequencies in other populations. A similar explanation was presented by Myers et al [10] and Rohlfs et al [12]. Second, there likely is a slight bias by using the same allele frequency data to generate the simulated profiles. True mother-child pairs were tested to investigate the effects of bias with the simulation strategy and to empirically confirm the findings by simulation studies. Table 2 shows the counts of minimum and maximum KI of the mother-child pairs distributed across the four major populations. Generally, consistent with the simulation studies, the minimum KI is closer to the true KI. The empirical data support that bias due to the simulation strategy is minor. The minimum KI should be considered as the selection criterion compared with the maximum KI as a strategy for familial searching.

**Table 1:** Accuracies of the minimum and maximum KIs being the true KI per population by simulations.

Population	Minimum KI				Maximum KI			
	True unrelated identified as		True Parent-Child	True Full-Sib	True unrelated identified as		True Parent-Child	True Full-Sib
	Parent-Child	Full-Sib			Parent-Child	Full-Sib		
African	82.1%	77.9%	74.9%	78.8%	4.6%	6.4%	8.0%	6.1%
Caucasian	64.1%	61.3%	59.1%	62.9%	2.3%	2.9%	3.0%	2.1%
SW Hispanic	73.7%	76.0%	65.4%	69.0%	2.9%	2.5%	5.3%	4.0%
SE Hispanic	38.0%	34.1%	37.2%	41.0%	1.2%	1.6%	1.3%	0.9%

For example, for true unrelated pairs identified as parent-child, if the true population is African American and four KIs are calculated with each of the four major populations, there is an 82.1% chance that the minimum KI is obtained with the true population (i.e., African American) and a 17.9% (i.e., 1–82.1%) chance that the minimum KI is obtained with any of the other populations. Likewise, for true parent-child pairs, if the true population is African American and four KIs are calculated with each of the four major populations, there is a 74.9% chance that the minimum KI is obtained with the true population (i.e., African American) and a 25.1% (i.e., 1–74.9%) chance that the minimum KI is obtained with any of the other populations.

**Table 2:** Counts of the minimum and maximum KIs of the mother-child pairs using empirical data.

Population	N	Minimum KI counts				Maximum KI counts			
		African	Caucasian	SW Hispanic	SE Hispanic	African	Caucasian	SW Hispanic	SE Hispanic
African	112	86	11	5	10	5	27	71	9
Caucasian	134	11	68	23	32	78	11	44	1
SW Hispanic	121	12	14	76	19	82	28	10	1

N is the total number of pairs per population. For example, in all 112 African American mother-child pairs, there are 11 pairs had the minimum KI associated with Caucasian population data.

a) Note that there was no empirical mother child pairs for southeastern Hispanics.

**Figure 2:**
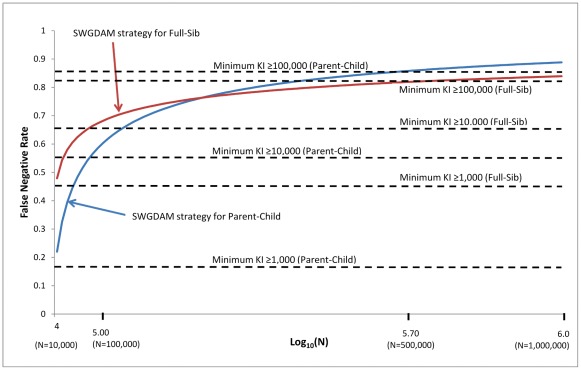
False negative rates of the SWGDAM recommendation 6 strategy and the minimum KI≥KI threshold strategy (i.e., KIs of 1,000, 10,000, and 100,000) based on 13 CODIS core loci and Caucasian population data for different sizes of databases from 10,000 to 1 million profiles. X axis is the log_10_(N); N is the database size. Y axis is the false negative rates (i.e., proportions of true relationships excluded) of the strategies.

**Figure 3:**
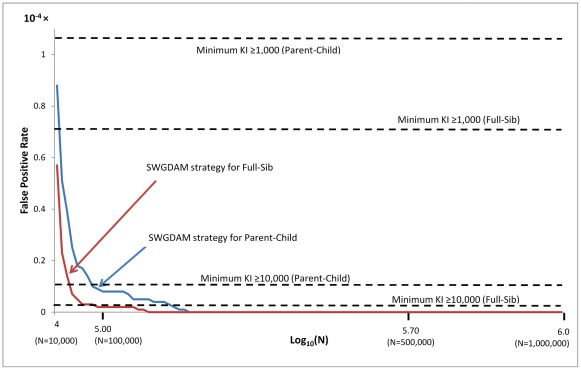
False positive rates of the SWGDAM recommendation 6 strategy and the minimum KI≥KI threshold strategy (i.e., KIs of 1,000 and 10,000) based on 13 CODIS core loci and Caucasian population data for different sizes of databases from 10,000 to 1 million profiles.

**Figure 4:**
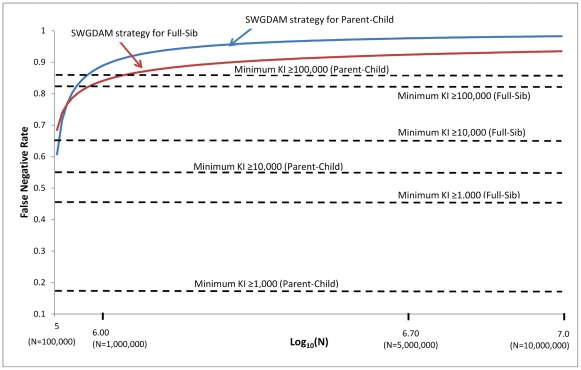
False negative rates of the SWGDAM recommendation 6 strategy and the minimum KI≥KI threshold strategy (i.e., KIs of 1,000, 10,000, and 100,000) based on 13 CODIS core loci and Caucasian population data for different sizes of databases from 100,000 to 10 million profiles.

The false negative and false positive rates of the SWGDAM strategy (i.e., recommendation 6) and the minimum KI≥KI threshold strategy were further compared. The KI threshold of the SWGDAM strategy varies with the database size, but the KI threshold of the minimum KI≥KI threshold strategy does not rely on the database size. Figures 2 and 3 show the false negative and false positive rates of these two strategies with pedigrees generated using Caucasian population data. For small databases with 10,000 profiles, the false negative rates of the SWGDAM strategy were 22.0% and 47.9% for Parent-Child and Full-Sib relationships, respectively, slightly higher than those of the minimum KI≥1,000 (Figure 2). With the same size database, the false positive rates of the SWGDAM strategy were 8.8×10^−5^ and 5.7×10^−5^ for Parent-Child and Full-Sib, respectively, slightly lower than those of the minimum KI≥1,000 (i.e. 1.1×10^−4^ and 7.0×10^−5^, respectively) (Figure 3). For a database with 100,000 or 1 million profiles, the false negative rates of the SWGDAM strategy were comparable with those of the minimum KI≥10,000 or ≥100,000, respectively. The false negative rates of the SWGDAM strategy were 88.8% and 83.9% for Parent-Child and Full-Sib relationships, respectively, with a database containing 1 million profiles, which were close to the false rates reported by Myers et al. [10] with three populations (i.e., 86% and 85%, respectively). The false negative rates were higher for larger databases, as expected. For a 10 million profile database, the false negative rates of SWGDAM strategy were 98.2% and 93.5% for Parent-Child and Full-Sib relationships, respectively (Figure 4). In other words, true relatives might be detected with less than 6.5% chance in a 10 million profiles database with the SWGDAM strategy. These high false negative rates suggest that the utility of familial searching for large databases, such as that of the current CODIS database with more than 10 million profiles, can be substantially reduced if a similar modest number of candidates is sought. On the other hand, even for a database with 100,000 profiles, the false positive rates of the SWGDAM strategy were extremely low to exclude most unrelated (and only include a few profiles). The EKR threshold of the SWGDAM strategy varies with the database sizes and could exclude the most offender profiles, unrelated or related, especially when the database size is large (e.g., more than 1 million). Therefore, for generating investigative leads, the SWGDAM recommendation 6 will be too stringent for large databases. Slooten et al. [13] also stated that there were good mathematical reasons not to regard the EKR as a good quantity measure of familial searching. The KI thresholds could be relaxed for large databases to include more offender profiles to increase the chance of placing a true relative (if in the database) on the candidate list without weakening the effectiveness of familial searching. Of course the candidate list can be subsequently filtered through Y-STR typing.

**Figure 5:**
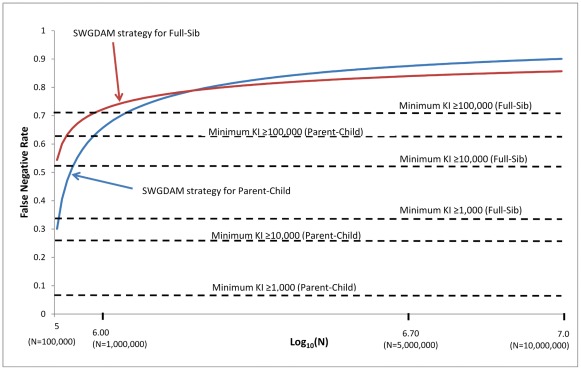
False negative rates of the SWGDAM recommendation 6 strategy and the minimum KI≥KI threshold strategy (i.e., KIs of 1,000, 10,000, and 100,000) based on 15 STR loci (i.e., 13 CODIS core loci, D2S1338, and D19S433) and Caucasian population data for different sizes of databases from 100,000 to 10 million profiles.

**Figure 6:**
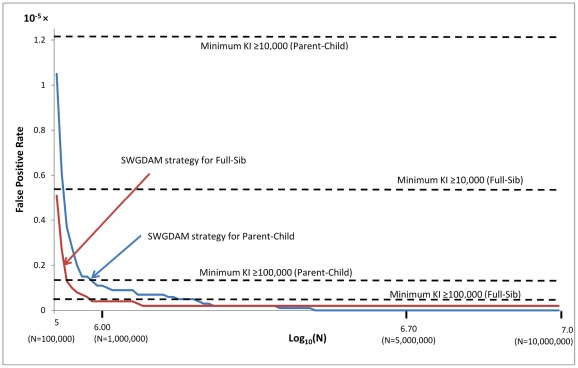
False positive rates of the SWGDAM recommendation 6 stragety and the minimum KI≥KI threshold strategy (i.e., KIs of 10,000 and 100,000) based on 15 STR loci (i.e., 13 CODIS core loci, D2S1338, and D19S433) and Caucasian population data for different sizes of databases from 100,000 to 10 million profiles.

California implemented the SWGDAM strategy for 13 CODIS core loci or 15 loci (i.e., 13 core loci, D2S1338, and D19S433) with three major populations (i.e., Caucasian, African American, and southwestern Hispanic) [10]. Adding extra STR loci to the 13 core loci changes the KI distributions. The effect is higher KIs for true relationships and lower KIs for unrelated pairs, reducing both false negative and false positive rates [11]. Similar simulations with 15 loci and three major populations, as in Myers et al [10], were performed herein. Figures 5 and 6 show the distributions of the false negative and positive rates of the SWGDAM strategy and the minimum KI≥KI thresholds with 15 STR loci. For a database with 1 million profiles, the false negative rates of the SWGDAM strategy were 65.8% and 72.3% for Parent-Child and Full-Sib relationships, respectively; the false positive rates of the SWGDAM strategy were 1.1×10^−6^ and 4.0×10^−7^ for Parent-Child and Full-Sib relationships, respectively. These values were comparable with those of the minimum KI≥100,000 and lower than the false negative rates with 13 loci. Both positive rates could be higher by decreasing the KI thresholds to include more offender profiles thereby reducing the chance of missing true relatives. With larger databases (e.g., 10 million profiles), the false negative rates of the SWGDAM strategy increase and the effectiveness of familial searching brought by additional loci will be diminished by the increased database size and the KI thresholds.

## Discussion

Likelihood ratio or kinship index based methods are more accurate than an IBS based method in kinship analysis and familial searching, because the allele frequency information is considered in a KI based method. However, when multiple relevant population affinities to which the forensic profile may potentially belong, multiple KIs will be calculated (one KI for each population). Thus, the KI cut-off threshold can impact the false positive and negative rates more so than when the scenario considers only one relevant population. There are generally three strategies for setting a KI threshold: (1) SWGDAM recommendation 6; (2) minimum KI≥KI threshold; and (3) maximum KI≥KI threshold. Since the minimum KI is more likely to be closer to the true KI, the maximum KI≥KI threshold may not be the best option. The KI thresholds of the SWGDAM strategy increase with the database size and the minimum and maximum KI thresholds are fixed when the database size is determined. However, the KI distributions are determined by the STR loci and their allele frequencies, not the database size. For large databases, the majority of offender profiles would be excluded from the candidate list with the SWGDAM strategy, regardless if related or unrelated. With a 1 million profile database, the chance to include a true Parent-Child or Full-Sib is only 11.2% and 16.1%, respectively, for the four US major populations, and this diminishes the effectiveness of familial searching to generate investigative leads. Myers et al. [10] showed high accuracies of identifying relatives based on both 13 or 15 autosomal STR loci and Yfiler® STR loci (i.e., 100% or 86% for parent-child and full-sib, respectively, with 15 loci) with 100 test families. The high accuracies, though, were mainly brought by the Y-STR loci. Without Y-STR loci, the accuracies significantly reduced to 28% or 38% for parent-child and full-sib, respectively [10]. Currently, the vast majority of offender profiles in the database do not contain Y-STR loci for direct searching and familial searching strategies. If all offender profiles contained a sufficient number of Y-STR loci, the familial searching strategies for autosomal STR loci may become less important for false positives, because after Y-STR screening, more than 99.9% of profiles would be excluded (the percent excluded will depend on the Y-STR loci used and population-specific haplotype frequencies). Moreover, if Y STR loci were contained within the database reference profiles, the KI threshold could be substantially reduced or even an IBS based method could be efficient for familial searching.

The minimum KI≥KI threshold strategy may be a better option for current autosomal STR loci based familial searching. The minimum KI strategy is better than the maximum KI strategy because the minimum KI generally is closer to the true KI. This observation is likely due to fact that the common profiles tend to have common or higher frequency alleles in their own population, which generally leads to lower KIs than the KIs calculated with other population data in which the same alleles may be less frequent. More importantly, in contrast with the SWGDAM strategy, the KI threshold can be adjusted according to resource demands, i.e., deciding false positive/negative rates and/or considering the size of the candidate list. Myers et al. [10] suggested choosing the top 168 offenders for further testing (in particular Y-STR typing), accommodated by two 96-well plates, which is a very practical and reasonable decision. The number of candidates using the SWGDAM strategy is fixed for a given size database and the forensic profile. Thus, the exact “168” is not necessarily related to some specified efficiency, but instead likely driven more so by resource constraints.

Indeed, the KI threshold is not particularly important if the number of candidates is pre-determined. However, one might consider that at least two candidate lists could be generated with multiple KI measures, i.e., KIs for parent-child or full-sib with 13 or 15 loci. The KI values from different relationships or different sets of markers cannot be compared because they are calculated under different frameworks. Suppose there is a target profile A to search against two offender profiles B and C, the KI of A and B favoring the parent-child hypothesis is 10 and the KI of A and C favoring the full-sib hypothesis is 20 (Equation 1).
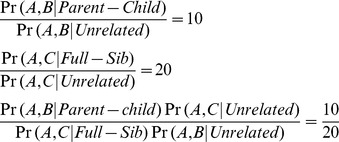
(1)


The data do not translate into that the full-sib scenario is more likely than the parent-child scenario, because 10/20 has no practical meaning. The same holds somewhat for comparisons of KIs calculated based on different sets of STR loci. The candidates should not be compared or ranked by the KI values generated from different frameworks (i.e., primarily relationship and somewhat by set of loci). More sophisticated approaches might be developed to merge the candidate lists from different frameworks. The profile-dependent or false rates based familial searching strategy proposed by Slooten et al. [13] could be a good approach to address this issue.

**Figure 7:**
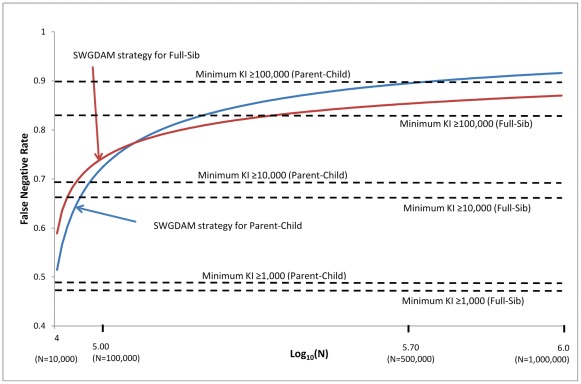
False negative rates of using jointly IBS≥16 and KI based methods, including the SWGDAM recommendation 6 strategy and the minimum KI≥KI threshold strategy (i.e., Kis of 1,000, 10,000 and 100,000), based on 13 CODIS core loci and Caucasian population data for different sizes of databases from 100,000 to 10 million profiles. The false negative rate is from 0.4 to 1 in the Y axis.

Both IBS and KI methods can be used jointly in familial searching practice, because the KI methods can not uniquely predict the IBS, and vice versa. Moreover, the IBS method is not dependent on population affinity (although the IBS distributions and confidence curves depend on population affinity), can reduce false positives, and is relatively simple to implement [11]. With an IBS threshold, the minimum KI is still the most likely KI to be closer to the true population. For example, with IBS≥16, about 60.3% and 64.2% of true Parent-Child and Full-Sib pairs had the minimum KI being the true KI for the Caucasian population, which is slightly higher than the proportions without an IBS threshold. Figure 7 shows the false positive and negative rates of using jointly IBS≥16 and various KI methods. As expected, joint use of IBS and KI can reduce the false negative rates. However, with higher KI thresholds for the minimum KI strategy or database size increasing for the SWGDAM strategy, the false negative rates with or without IBS approximate each other. Thus, using jointly IBS and KI does not significantly reduce the chance to include the true relative into candidate list for large databases, but does provide a higher efficiency, determined by success rate, for familial searching.

In summary, current familial searching strategies are developed mainly based on autosomal STR loci. Y STR data are not used initially because most of the offender profiles in the databases do not have Y-STR (or mitochondrial DNA) data. The SWGDAM strategy may be too stringent for familial searching for large databases (e.g., 1 million or more profiles). The minimum KI≥KI threshold strategy apparently is a better option, which provides the flexibility to adjust the KI threshold according to the pre-determined number of candidates or false positive/negative rates.
